# Immunoinformatic and systems biology approaches to predict and validate peptide vaccines against Epstein–Barr virus (EBV)

**DOI:** 10.1038/s41598-018-37070-z

**Published:** 2019-01-24

**Authors:** Arif Ali, Abbas Khan, Aman Chandra Kaushik, Yanjie Wang, Syed Shujait Ali, Muhammad Junaid, Shoaib Saleem, William C. S. Cho, Xueying Mao, Dong-Qing Wei

**Affiliations:** 1 State Key Laboratory of Microbial Metabolism, and College of Life Sciences and Biotechnology, Shanghai Jiao Tong University, Shanghai, China; 2 Center for Biotechnology and Microbiology, University of Swat, Khyber Pakhtunkhwa, Pakistan; 3 Department of Clinical Oncology, Queen Elizabeth Hospital, Kowloon, Hong Kong; 4 Qianweichang College, Shanghai University, Shanghai, China

## Abstract

Epstein–Barr virus (EBV), also known as human herpesvirus 4 (HHV-4), is a member of the Herpesviridae family and causes infectious mononucleosis, Burkitt’s lymphoma, and nasopharyngeal carcinoma. Even in the United States of America, the situation is alarming, as EBV affects 95% of the young population between 35 and 40 years of age. In this study, both linear and conformational B-cell epitopes as well as cytotoxic T-lymphocyte (CTL) epitopes were predicted by using the ElliPro and NetCTL.1.2 webservers for EBV proteins (GH, GL, GB, GN, GM, GP42 and GP350). Molecular modelling tools were used to predict the 3D coordinates of peptides, and these peptides were then docked against the MHC molecules to obtain peptide-MHC complexes. Studies of their post-docking interactions helped to select potential candidates for the development of peptide vaccines. Our results predicted a total of 58 T-cell epitopes of EBV;  where the most potential were selected based on their TAP, MHC binding and C-terminal Cleavage score. The top most peptides were subjected to MD simulation and stability analysis. Validation of our predicted epitopes using a 0.45 µM concentration was carried out by using a systems biology approach. Our results suggest a panel of epitopes that could be used to immunize populations to protect against multiple diseases caused by EBV.

## Introduction

Epstein–Barr virus (EBV), also known as human herpesvirus 4 (HHV-4), is a member of the Herpesviridae family and is one of the eight known types of human herpesvirus. EBV is the most common human virus in the world^1^ and was isolated in 1964 from tumor cells (Burkitt’s lymphoma) by Epstein’s group^2^. EBV is related to distinct forms of cancer, such as Burkitt’s lymphoma, stomach cancer, Hodgkin’s lymphoma and nasopharyngeal carcinoma^3,4^. A High number of cases are usually reported. In the United States and other developing countries, most people are infected with EBV^5^ as 90% of the adults in the United States have been formally diagnosed with EBV infection. EBV infection can be asymptomatic or symptomatic, and the latter case includes mild fatigue, fever, enlarged spleen, swollen liver, swollen lymph nodes, inflamed throat, or rashes^6^. From 2006 to 2015, several clinical trials were conducted to develop vaccines; however, an EBV vaccine, phase 2 trial, from gp350 protein has been testified. This vaccine reduced the rate of Infectious Mononucleosis (IM) but not virus infection^7^.

Precautionary measures, such as avoiding direct contact with patients (including refraining from using a patient’s toothbrush, sharing food, or exchanging bodily fluids), can help reduce the risk of infection. EBV can infect host B cells and booms via a nonlytic mechanism^8^. EBV viral proteins play important roles in lymphoproliferative disease. For example, viral membranous proteins, such as LMP-1, may induce tumorigenic replication in infected B cells^9,10^. There are two types of EBV; Epstein and Yvonne Barr identified EBV in tumor tissue associated with Burkitt’s Lymphoma^11^. Glycoprotein 42 (gp42), Glycoprotein H, Glycoprotein L, and Glycoprotein B aid in the entrance to the host cell. Glycoprotein 42 binds to the HLA class II molecule because it is required for B cells, which inhibit epithelial cell fusion. For epithelial-cell fusion, the GH receptor protein interacts with GP42^12^ and GL is transported to the cell surface, which is essential for the correct folding of GH^13^.

EBV glycoprotein B is important for viral fusion events with B cells^14^. The human immune system precisely targets EBV glycoprotein 350 (gp350), which is an example of a lytically expressed gene^15^. The attachment of GP350 to the MHC-II molecules in the cell is aided by the already attached GP42 protein of EBV virus^16^. The fusion of the B-cell membrane and the outer viral envelope of the EBV virion requires functional spicule glycoproteins such as GH, GL, and gp42^17^. Previous studies suggest that glycoproteins such as GB complement membrane fusion^18^.

Vaccination is a significant approach to improve the standard of public health and provide an effective way to control the growing infections. In nature, plants act as bioreactors, which have been used to express efficient vaccine antigens against viral, bacterial and protozoan infections. Besides, we know that antibody epitope prediction using computational tools, one of the crucial steps of vaccine design^19^. Recent advancement in vaccine design has aimed for the expansion of conventional assays designed to quantify T-cell responses against various vaccine candidates^20^. Immunoinformatic approaches have made great contributions to predicting B-cell and T-cell epitopes in the development of subunit vaccines^21^. For subunit vaccine development, identification of continuous B-cell or nonlinear also known as non-continuous and cytotoxic T-lymphocyte (CTL) epitopes are essential. Among B-cell epitopes, >90% are noncontinuous^22,23^. The use of computational tools contribute greatly in biology designing *in silico* vaccine, prediction of T-cell epitope is crucial which does not only reduce the cost but also the necessity for experimental results^24^. Epitopic vaccine against HIV, malaria and tuberculosis resulted in promising outputs and maintained the defensive and beneficial potential therapeutic uses of the developed vaccines candidates^25^. Immunoinformatics plays an upright role in antibody and immunodiagnostic agents development, and vaccine design. The early phases of vaccine and other therapeutics agents developments were based on solely immunological experimentations. These early developmental techniques were tedious and costly too. The use of bioinformatics such as computational techniques greatly reduces the time and cost of developing such agents for therapeutic purposes. Khan *et al*.^26^, used multiple bioinformatics tools to predict vaccine against multiple HPV viruses^26^. Thus the development of modern therapeutic medicines and vaccines greatly rely on such tools^27,28^.

Systems medicine emphases significantly on the components of pathway kinetics to probe different conditions. Systems medicine could be also utilized to investigate interaction mechanisms between microbes. Metagenomics data could be utilized for such analysis. Rather than whole cell interaction, an insight onto proteins interaction could be also comprehended through systems biology approaches (Singh, P. K. *et al*.^29^. These latest techniques widely expand the circle of new drugs development. Overall, it is known that multidisciplinary aspects of the production of therapeutic proteins that has gained much more attention (Dangi, A. K. *et al*.^30^. Structural modelling using computational resources led to success in the development of computer assisted drugs. Likewise molecular docking algorithms scoring functions of different conformers to design new drug candidates.

In the present study, potential B-cell and T-cell epitopes (as effective vaccine candidates) were identified with the help of immunoinformatic approaches. T-cell immunogenicity is associated with epitope binding strength to the MHC molecule^31^. Molecular modelling tools were applied to peptide-MHC complexes to investigate their post-docking interaction in order to select potential candidates for the development of peptide vaccines.

## Materials and Methods

### Epstein-Barr Virus Sequence

**Figure 1:**
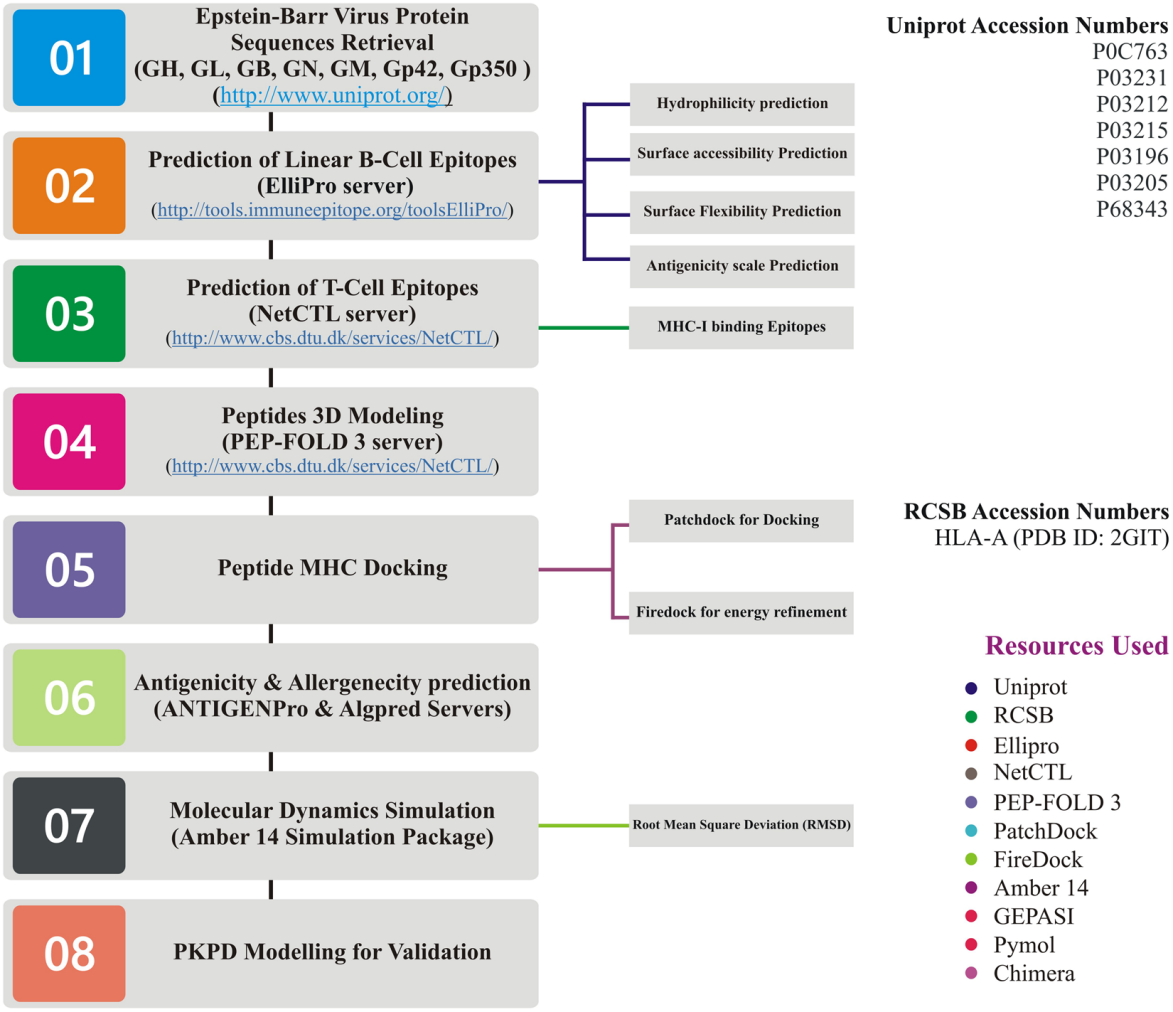
The figure above is showing the pipeline of the study. Resources, methods, and each step is discussed.

The sequences of EBV proteins, including GH, GL, GB, GN, GM, GP42 and GP350, were retrieved from the Universal Protein knowledgebase (Uniprot) database (http://www.uniprot.org/). The sequence retrieval accession numbers along with other information are provided in the (Table 1). The 3D coordinates of all the selected proteins were predicted by using online webserver phyre2 (http://www.sbg.bio.ic.ac.uk/phyre2/html/page.cgi?id = index)^32^. The overall workflow of the work is shown in the Fig. 1.

**Table 1:** Detailed information, including individual protein sequence length, and region and accession number is shown in the table below.

S. No	Uniprot Accession Number	Protein Name	No of Amino Acids
1.	P0C763	Glycoprotein B	857
2.	P03231	Glycoprotein H	706
3.	P03212	Glycoprotein L	137
4.	P03215	Glycoprotein M	405
5.	P03196	Glycoprotein N	102
6.	P03205	Glycoprotein 42	223
7.	P68343	Glycoprotein 350	886

### Prediction of Linear B-Cell Epitopes

After interacting with antigens (such as B-cell epitopes), B-lymphocyte cells differentiate into memory cells and antibody secreting plasma cells^33^. B-cell epitopes have a hydrophilic nature and are accessible for flexible regions^34^. IEDB (http://www.iedb.org/) online analysis resources were used to obtain the Parker hydrophilicity prediction values^35^, Emini prediction values of surface accessibility^36^, Kolaskar and Tongaonkar’s antigenicity scale values^37^, and Karplus and Schulz Flexibility Prediction values. B-cell epitopes were predicted using ElliPro (http://tools.immuneepitope.org/toolsElliPro/) using both protein sequences and structural information^38^. ElliPro utilizes the Protrusion Index (PI) of residues, protein shape approximation, and the final neighbouring residues clustering, which rely on PI.

### Prediction of Potential Cytotoxic T-lymphocyte (CTL) Epitopes

CTL epitopes were predicted using the NetCTL.1.2 (http://www.cbs.dtu.dk/services/NetCTL/) server^28^. NetCTL accepts the FASTA sequence format to perform different analyses, such as prediction of MHC class I binding affinity, TAP transport efficiency and C-terminal cleavage. The artificial neural network and weight matrix were used for the prediction of MHC-I binding and proteasome-dependent C-terminal cleavage.

### Peptide Library Construction and Molecular Docking

All of the predicted epitopes were modelled using the online webserver PEP-FOLD3 using 200 simulation runs to sample the conformations^39^ and sOPEP energy function^40^. Subsequently, we have docked the best ranked peptide models to the selected class I MHC molecules HLA-A (PDB ID: 2GIT) using the PatchDock docking server^41^. The algorithm of the PatchDock server uses structural geometry to find docking transformations with good molecular shape complementarity^42^. The resulting complexes were refined through the FireDock server^43,44^. High energy complexes were subjected to interaction analysis and molecular dynamics simulations^45^.

### Molecular Dynamics Simulations

The accepted complexes were subjected to Molecular Dynamic simulations using the AMBER 14 molecular dynamics package^46^. The system was neutralized using Na^+^ ions using tleap. Each system was solvated in a rectangular box with buffer distance of 8.0 A° using TIP3P water molecules. A two-stage energy minimization of the complexes, using the SANDER module of AMBER 14, was performed to relieve the atomic clashes. An initial minimization of 6,000 steps, followed by another round of minimization (6,000 steps), were used to restrain the positions of all atoms in the systems, except those from the water molecules in the first minimization. The *pmemd.cuda*^47^ software was used to simulate the minimized complexes. The SHAKE algorithm and the Particle-Mesh Ewald (PME) method were used to include the long-range interactions, and a non-bonded interaction cutoff radius of 10 A° was considered. For equilibration, 10,000 ps time was applied, followed by a 50 ns simulation carried out at 310 K using the Langevin temperature coupling scheme at constant pressure (1 atm) with isotropic molecule-based scaling. Sampling of the MD trajectories was carried out every 2.0 ps. RMSD and hydrogen bonding analysis were carried out using the integrated CPPTRAJ and PYTRAJ^48^ modules in AMBER 14 and were visualized using the online server PDBePISA^49^, UCSF Chimera^50^ and PyMOL^51^.

### Antigenic and Allergenic behaviour the predicted Epitopes

To confirm the allergenic and non-allergenic properties of all the designed epitopes, B-cell and T-cell epitopes, AlgPred^52^ (http://crdd.osdd.net/raghava/algpred/), which is an online web tool was employed with accuracy of 85%. Primary amino acid sequences were used of all the selected proteins for this purpose. The antigenicity of all the epitopes was predicted by using ANTIGENpro^53^ (http://scratch.proteomics.ics.uci.edu/) using different machine learning algorithms to process the amino acids sequences.

### PKPD Modeling

The design and execution of the EBV signalling cascade was performed based on a literature survey within a virtual cell. Proposed peptides were chosen for kinetic analysis of EBV, where a concentration of 0.45 µm was assigned based on previous literature. Modelling of chemical reaction networks was applied for the analysis of this pathway.

This nonlinear kinetics scheme follows the Michaelis–Menten equation as below:

1 $${\rm{V}}=\frac{({\rm{Vmax}})\,\cdot \,({\rm{S}})}{{\rm{Km}}+{\rm{S}}}$$

This equation can be transformed to,

$${\rm{C}}=\frac{({\rm{Cmax}})\,\cdot \,({\rm{D}})}{{\rm{Km}}+{\rm{D}}}$$

or

2 $${\rm{V}}=\frac{{\rm{d}}[{\rm{P}}]}{{\rm{dt}}}=\frac{({\rm{Amax}})\,\cdot \,({\rm{D}})}{{\rm{Km}}+{\rm{D}}}$$

Here,

C = steady state concentration;

C_max_ = theoretical maximum for C;

A_max_ = theoretical maximum for A;

D = dose.

## Results

### Sequence retrieval and analysis

**Figure 2:**
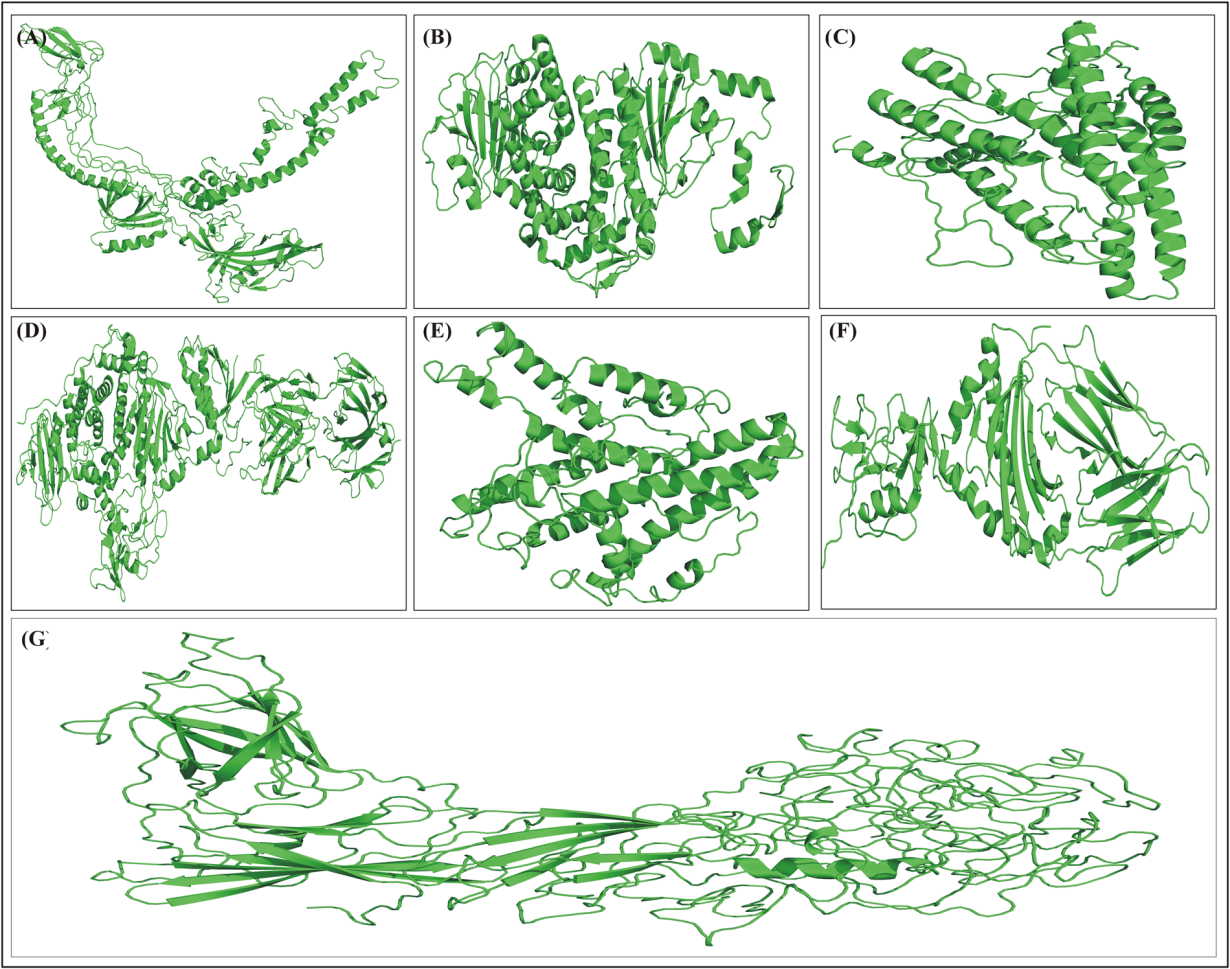
The 3D structures of the selected proteins. The Phyre 2 online server was used for B-cell and T-cell epitope prediction. (**A**) Glycoprotein B (**B**) Glycoprotein H (**C**) Glycoprotein L (**D**) Glycoprotein M (**E**) Glycoprotein N (**F**) Glycoprotein 42 (**G**) Glycoprotein 350.

Uniprot was used to retrieve the primary amino acid sequences of the selected proteins (glycoprotein B, glycoprotein L, glycoprotein N, glycoprotein H, glycoprotein M, glycoprotein 42 and glycoprotein 350) of EBV as shown in Fig. 2. Information about the protein source, accession number, number of active residues, and other information is given in Table 1.

### Allergenicity and antigenicity prediction

The allergic and nonallergenic behaviours of EBV species were predicted using AlgPred (http://www.imtech.res.in/raghava/algpred/). Allergenicity prediction of known protein sequences is based on similarity. We checked if the epitopes are antigenic or not using the online server AntigenPro (http://www.scratch.proteomics.ics.uci.edu/)^35^. All of the proteins were found to be nonallergenic, while they possess antigenic properties (Table 2).

**Table 2:** Antigenic and allergenic results of the selected proteins.

S.NO	Proteins	Allergenicity	Antigenicity
1.	Glycoprotein B	Non-Allergic	Antigenic
2.	Glycoprotein H	Non-Allergic	Antigenic
3.	Glycoprotein L	Non-Allergic	Antigenic
4.	Glycoprotein M	Non-Allergic	Antigenic
5.	Glycoprotein N	Non-Allergic	Antigenic
6.	Glycoprotein 42	Non-Allergic	Antigenic
7.	Glycoprotein 350	Non-Allergic	Antigenic

### B-cell epitope prediction

The BCPred server predicted 58 B-cell epitopes: five epitopes were for GP 42, eight for GP H, nineteen for GP B, one for GP L and GP N, five for GP M, and nineteen for GP 350. However, epitopes with scores above 0.99 were selected as the most potentially antigenic epitopes. Therefore, only one epitope each from GP42, GL, GM, GN and GH; four epitopes from GB; and fifteen epitopes from GP350 were found to meet the threshold value. B-cell epitopes, along with their scores, are tabulated in Table 3.

**Table 3:** B-Cell epitopes predicted by BCPred.

Protein	Position	Epitope	Score
GP42	43	TWVPKPNVEVWPVDPPPPVN	1
GH	623	DEKEGLETTTYITSQEVQNS	0.994
GB	21	GAQTPEQPAPPATTVQPTAT	1
	400	TTPTSSPPSSPSPPAPSAAR	1
	430	RRRDAGNATTPVPPTAPGKS	1
	257	YKIVDYDNRGTNPQGERRAF	1
GL	18	LPTWGNWAYPCCHVTQLRAQ	0.674
GM	357	TPSPGRNRRRSSTSSSSSRS	1
GN	25	TGVLPAGASSPTNAAAASLT	1
GP350	514	TTPTPNATSPTPAVTTPTPN	1
	535	TSPTPAVTTPTPNATSPTLG	1
	474	TSPTPAGTTSGASPVTPSPS	1
	598	TSPTSAVTTPTPNATGPTVG	1
	720	PAPRPGTTSQASGPGNSSTS	1
	577	TSPTSAVTTPTPNATSPTLG	1
	556	TSPTSAVTTPTPNATSPTLG	1
	423	KAPESTTTSPTLNTTGFADP	1
	835	TSPPVTTAQATVPVPPTSQP	1
	647	TSAVTTGQHNITSSSTSSMS	1
	243	GILTSTSPVATPIPGTGYAY	1
	452	THVPTNLTAPASTGPTVSTA	1
	626	TNHTLGGTSPTPVVTSQPKN	1
	746	NVTKGTPPQNATSPQAPSGQ	1
	767	TAVPTVTSTGGKANSTTGGK	1

### Surface accessibility of EBV

Threshold values >1 were set to predict the surface probability values. Amino acids with higher surface probability values (>1) have greater probability to be present on protein surfaces^36^. The maximum surface probability scores for Glycoprotein B (RRRRRD_428–433_), Glycoprotein H (EREDRD_520–525_)_,_ Glycoprotein L (KNGSNQ_68–73_), Glycoprotein M (RNRRRS_362–367_), Glycoprotein N (TEAQDQ_44–49_), Glycoprotein 42 (TKKKHT_199–124_), and Glycoprotein 350 (PRPRYN_810–815_) were 9.415, 9.265, 4.395, 9.054, 4.777, 5.691 and 4.859, respectively. The minimum surface probability scores were 0.032 (VVILVI_745–750_), 0.033 (CVFCLV_5–10_), 0.071 (LAICLV_8–13_), 0.067 (IIPILC_309–314_), 0.07 (LVLVII_76–81_), 0.054 (VIVLLL_18–23_), and 0.058 (AALLVC_3–8_). Figure S1 (Supplementary Materials) shows the graphical representation of the predicted surface accessibility of EBV. Moreover, for all of the other proteins which have maximum and minimum accessibility scores are shown in Table S1 (Supplementary Materials).

### Surface flexibility of EBV selected proteins

The Karplus and Schulz flexibility method was used to calculate the motions of atoms (back and forth, considering temperature or B factor). Low B-factor values indicate a highly systematic structure, and high B factors indicate a distorted structure 32. The graphical representation of the surface flexibility results for EBV is shown in Fig. S3. Maximum flexibility scores for Glycoprotein B, Glycoprotein H, Glycoprotein L, Glycoprotein M, Glycoprotein N, Glycoprotein 42, and Glycoprotein 350 were 1.13, 1.094, 1.121, 1.157, 1.071, 1.091, 137 for heptapeptides EQNQEQK_803–809,_ VITQGPN_346–442_, PKNGSNQ_67–73_, STSSSSS_368–374_, GASSPTN_31–37_, VRGGGRV_31–37_, PGNSSTS_733–739_, respectively. Minimum flexibility scores were 0.88, 0.872, 0.904, 0.866, 0.862, 0.897, and 0.894 for the peptides QAIMLAL_795–801_, LAAMLMA_351–357_, FLAICLV_7–13_, FLWWVVF_193–199_, IYLMYVC_85–91_, VAAAAIT_37–43_, and LLVMADC_877–883_, respectively. Figure S2 (Supplementary Materials) shows a graphical representation of the predicted surface flexibility of EBV. Moreover, for all of the other proteins which have maximum and minimum flexibility scores are shown in Table S2 (Supplementary Materials).

### Parker Hydrophilicity Prediction for EBV

Hydrophilicity of the predicted epitopes was calculated using the Parker hydrophilicity approach^35^. The graphical illustration of the predicted Parker hydrophilicity of EBV is shown in Fig. S3 (Supplementary Materials). From all of the predicted EBV peptides, the maximum hydrophilicity calculated was 6.843 for Glycoprotein 350 at the amino acid positions DNGTESK_496–502_. These regions were predicted to act as active T-cell epitopes. The minimum hydrophilicity score calculated was −7.857 for Glycoprotein M at the amino acid positions FLWWVVF_193–199_. Moreover, for all of the other proteins which have maximum and minimum hydrophilicity scores are shown in Table S3 (Supplementary Materials).

### T-cell epitope identification

Epitope predictions for all seven proteins were conducted on NetCTL, an online epitope prediction server. MHC-I binding prediction using the SMM method resulted in many potential epitopes against one allele, HLA-A*24:02. The weight matrix and artificial neural network was used for the prediction of MHC-I binding and proteasome dependent C-terminal cleavage. The MHC binding affinity, the TAP score, and the C-terminal cleavage score were considered to select the most promising epitopes among those predicted. The top three epitopes (as shown in Table 4) for glycoprotein B (ETDQMDTIY, QMDTIYQCY, PTTVMSSIY), glycoprotein L (MTAASYARY, LTSAQSGDY, ATSVLLSAY), glycoprotein H (ALENISDIY, LLTTLETLY, SSSALTGHL), glycoprotein N (IADCVAFIY, FLALGNSFY, TTDSEEEIF), glycoprotein M (LTEAQDQFY, IASAIYLMY, ASAIYLMYV), glycoprotein 42 (CAELYPCTY, HTFQVPQNY, NTREYTFSY), and glycoprotein 350 (PTNTTDITY, FLGNNSILY, HAEMQNPVY) were selected for docking.

**Table 4:** List of the total peptides T-cell vaccines predicted by NetCTL.

	S.NO	Peptide Sequence	MHC Binding Affinity	Rescale Binding Affinity	C-terminal Cleavage Affinity	Transport Affinity	Prediction Score	MHC-I Binding
GB	131	ETDQMDTIY	0.7945	3.3733	0.6371	2.4710	3.5924	Yes
	134	QMDTIYQCY	0.7028	2.9841	0.9533	2.7440	3.2643	Yes
	502	PTTVMSSIY	0.5331	2.2633	0.6136	2.5280	2.4817	Yes
GL	256	MTAASYARY	0.7300	3.0993	0.6470	2.9770	3.3452	Yes
	216	LTSAQSGDY	0.7246	3.0764	0.6687	2.9570	3.3245	Yes
	396	ATSVLLSAY	0.6201	2.6327	0.9581	3.0130	2.9271	Yes
GH	41	ALENISDIY	0.4625	1.9636	0.9238	2.9920	2.2518	Yes
	107	LLTTLETLY	0.3076	1.3059	0.9638	2.7450	1.5877	Yes
	96	SSSALTGHL	0.1437	0.6103	0.9131	1.1760	0.8060	Yes
GN	86	IADCVAFIY	0.6262	2.6588	0.9587	2.7190	2.9385	Yes
	225	FLALGNSFY	0.5351	2.2720	0.8655	2.9130	2.9385	Yes
	396	TTDSEEEIF	0.4510	1.9148	0.2635	2.3520	2.0719	Yes
GM	43	LTEAQDQFY	0.7612	3.2319	0.6749	2.8170	3.4740	Yes
	81	IASAIYLMY	0.5205	2.2100	0.7658	3.0050	2.4752	Yes
	82	ASAIYLMYV	0.1686	0.7159	0.5794	0.5250	0.8291	Yes
GP42	132	CAELYPCTY	0.4738	2.0117	0.9710	2.9460	2.3047	Yes
	86	HTFQVPQNY	0.4422	1.8777	0.9494	2.9720	2.1687	Yes
	103	NTREYTFSY	0.3707	1.5740	0.9745	2.9820	1.8693	Yes
GP350	316	PTNTTDITY	0.5572	2.3659	0.9498	2.3650	2.6266	Yes
	274	FLGNNSILY	0.4340	1.8426	0.9710	2.8180	2.1291	Yes
	143	HAEMQNPVY	0.4028	1.7103	0.7631	2.7190	1.9607	Yes

### Peptide modelling and docking studies for HLA-A*24:02 and epitope interaction analysis

**Figure 3:**
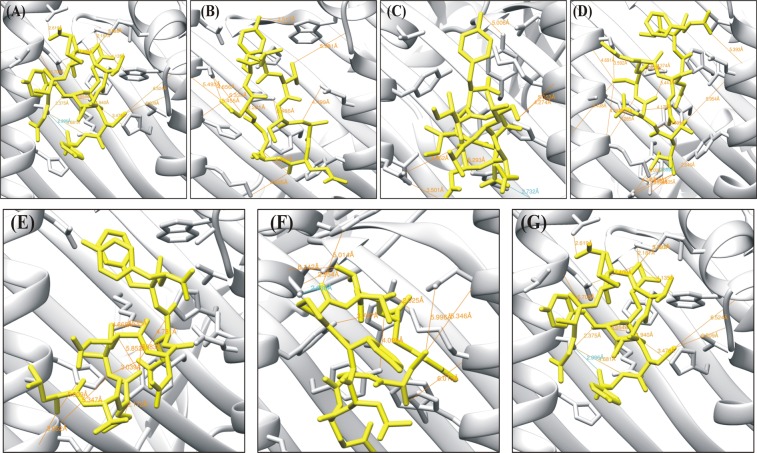
Interaction pattern of the docked peptides against MHC I molecules.

The selected top three epitopes from all proteins were docked against HLA-A*24:02 using Fire dock. The epitope QMDTIYQCY (glycoprotein B) was docked to understand the interaction pattern between peptide-MHC complexes. The global docking energy and van der Waals (vdW) energy were reported as −35.20 (kcal/mol) and −25.12 (kcal/mol), respectively. Residues Gln7, Cys8 and Asp3 from the docked peptides and Thr143, Tyr116, Thr80 and Lys146 from the MHC molecules were involved in binding. Peptide MTAASYARY (glycoprotein H) was reported to share a global energy of −34.27 (kcal/mol), with −29.12 (kcal/mol) vdW energy. On the other hand, peptides from Glycoprotein M (TTDSEEEIF), Glycoprotein N (LTEAQDQFY), Glycoprotein 42 (CAELYPCTY), and Glycoprotein 350 (PTNTTDITY) contributed global energies of −36.20 (kcal/mol), −34.25 (kcal/mol), −40.20 (kcal/mol), and −30.48 (kcal/mol), respectively. The vdW interaction energies for these complexes ranged from −29.71(kcal/mol) to −18.80 (kcal/mol). Residues Lys66, Arg97, Tyr99, Gln155, His114, and Thr163 from these complexes were uniformly involved in hydrogen bonding interactions. The Chimera interaction analysis tool predicted the interactions between peptide and MHC-I molecules within 3Ǻ. Overall, the stability was supported by the variable amounts of hydrogen bonding. The molecular interaction patterns are depicted in Fig. 3, while the interacting atoms are shown in Table 5.

**Table 5:** Molecular Docking analysis of the final peptides.

Peptide	Global Energy (kcal/mol)	vdW Energy (kcal/mol)	H-Bond Energy (kcal/mol)	H-Bond Interaction	
				Peptide-MHC atom pair	d_init_ (Å)
QMDTIYQCY	−35.20	−25.12	−1.09	GLN7 NE2-THR143 OG1	3.39
Glycoprotein B				CYS8 N-TYR116 OH	3.77
				ASP3 OD1-THR80 OG1	2.62
				ASP3OD2-LYS146 NZ	3.95
MTAASYARY	−34.27	−29.12	−3.82	GLN5 NE2-GLU63 O	3.35
Glycoprotein H				GLN5 NE2-GLU63 OE2	3.60
				SER6 N-TYR99 OH	3.47
				TYR9 N-GLN155 OE1	2.42
				THR2 O-THR73 OG1	3.56
				THR2 O-ARG97 NH1	3.63
				SER3 OG-HIS70 NE2	3.01
				GLN5 OE1-TYR99 OH	3.77
				ASP8 OD2-HIS114 NE2	3.79
				ASP8 OD2-ARG97 NH2	3.76
MTAASYARY	−34.26	−18.80	−1.36	GLN5 NE2-THR163 OG1	2.74
Glycoprotein H				SER6 O-TYR99 OH	2.56
				GLY7 O-ARG7 NH1	2.22
				TYR9 OH-TRP147 NE1	3.52
TTDSEEEIF	−36.20	−29.71	−4.82	THR1 OG1-GLU63 OE2	3.03
Glycoprotein M				THR1 O-LYS66 NZ	2.89
				GLU7 OE1-ARG97 NH2	3.72
				GLU7 OE2-ARG97 NE	3.69
				GLU7 OE2-ARG97 NH2	3.02
				GLU7 OE2-HIS114 NE2	3.24
LTEAQDQFY	−34.25	−24.49	−3.30	LEU1 N-ARG65 O	3.65
Glycoprotein N				GLU3 OE2-TYR99 OH	2.17
				ASP6 O-LEU156 N	3.88
				GLN7 OE1-HIS70 NE2	3.04
				GLU3 OE1-LYS66 NZ	3.60
				GLU3 OE2-HIS70 NE2	3.32
CAELYPCTY	−40.20	−23.44	−1.23	CYS7 N-THR163 OG1	3.50
Glycoprotein 42				THR8 OG1-THR163 O	2.95
				TYR9 N-TYR159 OH	3.74
				GLU3 O-GLN155 NE2	3.13
				PRO6 O-THR163 OG1	2.03
				THR8 O-LYS66 NZ	2.02
PTNTTDITY	−30.48	−29.45	−3.12	THR5 OG1-THR143 OG1	3.48
Glycoprotein 350				ASN3 OD1-TRP147 N	3.54
				THR4 O-THR80 OG1	3.58
				THR4 OG1-LYS146 NZ	2.53
				ASP6 O-TRP147 NE1	3.57
				ASP6 OD2-ARG97 NH2	3.50
				ASP6 OD1-ARG97 NH2	3.89
				ASP6 OD2-ARG97 NH2	3.50
				ASP6 OD2-HIS114 NE2	3.27

### PKPD modelling-based validation

**Figure 4:**
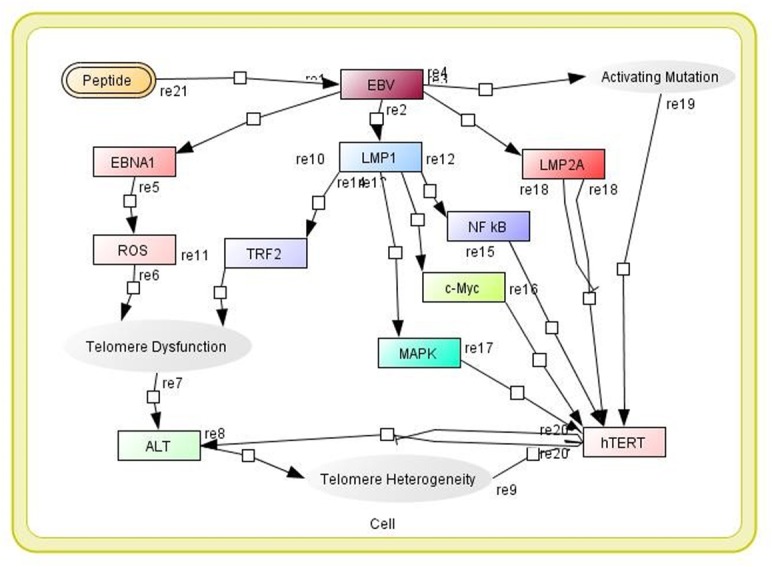
Model of the biochemical pathway of MDS in the presence of peptides.

**Figure 5:**
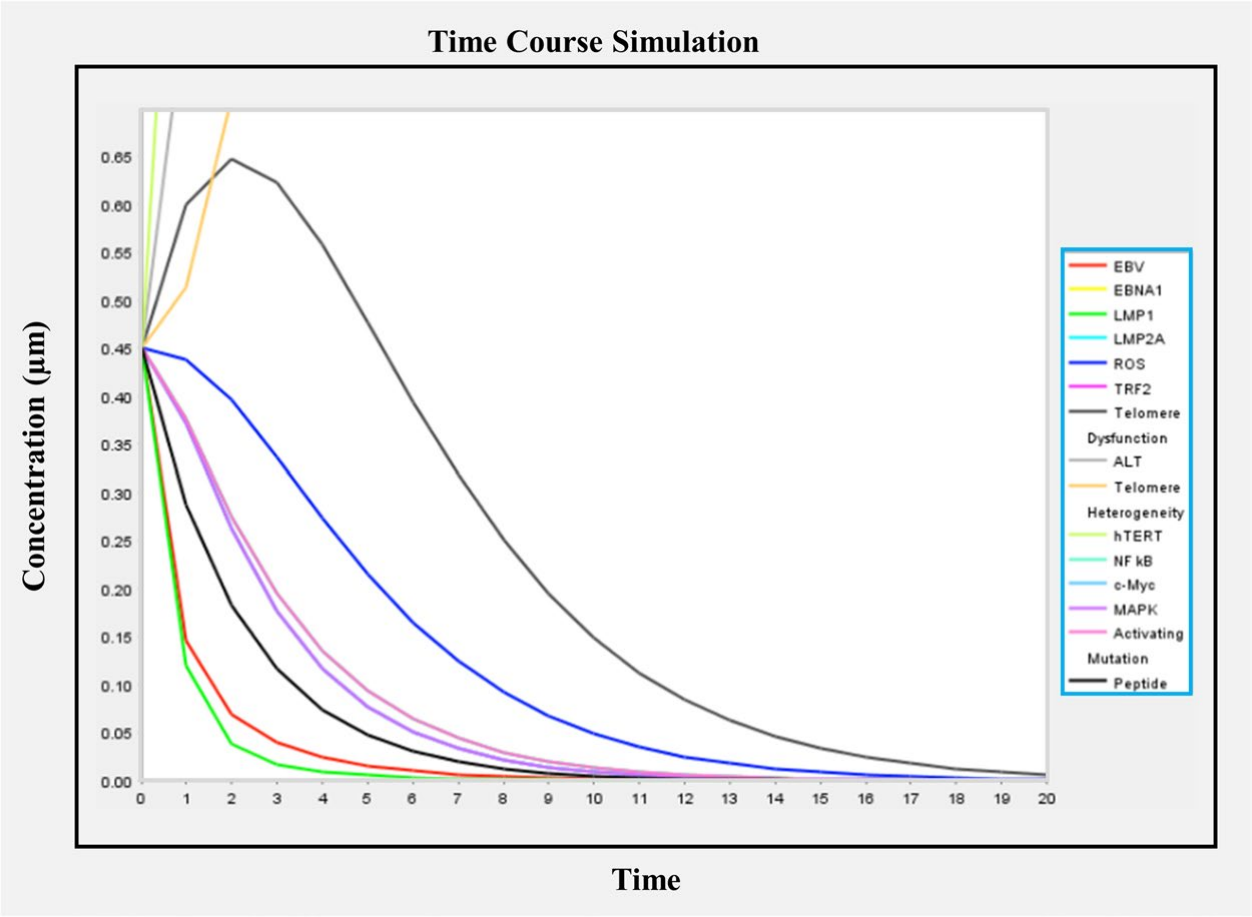
The time-course simulation with screened peptides against relation of interacting EBV.

In a time-course simulation with the shortlisted peptides (obtained from the screening), the initial concentration of the top-listed peptides was set at 0.45 µm^54–56^, and after 20 seconds, all of the interactions of the EBV mechanism in the cell signalling cascade were stabilized given in Fig. 4 and 5. EBV is a Baltimore Class I virus of the Herpesviridae family and plays a crucial role in lymphoproliferative disease. During tropic EBV infection, EBV binds to the HLA class II molecule, and B cells inhibit epithelial cell fusion, while the GH receptor protein interacts with GP42, and GL is transported to the cell surface where it is essential for the correct folding of GH. EBV GB is important for viral fusion events with B cells. Glycoprotein 350 binding is supported by the binding of EBV gp42 to B-cell MHC-II, while fusion of the B-cell membrane and the outer viral envelope of EBV virion must have functional spicule glycoproteins, such as GH, GL and gp42. Vaccines development requires the identification of extremely competent B-cell linear or nonlinear and CTL epitopes, where T cells act as mediators.

A systems biology approach is useful to investigate T-cell epitopes in peptide sequences, and earlier reports have accelerated research leading to the development of immune biology.

### Root Mean Square Deviation (RMSD)

**Figure 6:**
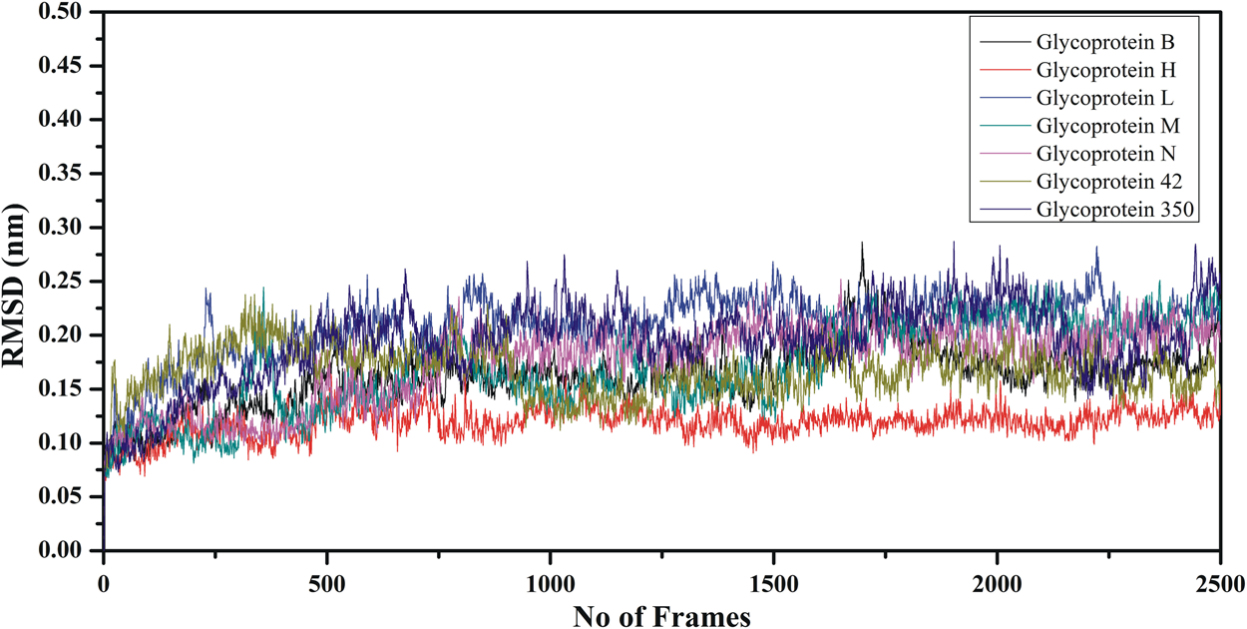
Root mean square deviation (RMSD) of the peptide-MHC complexes showing the stability of the peptides in the binding cavity of the MHC molecule.

Molecular dynamics simulation was conducted to confirm the post-docking stability of the complexes. Trajectories were obtained after 50 ns and subjected to backbone stability using RMSD. RMSD of the selected complexes after 50 ns revealed that all of the complexes were stable and that the peptides had occupied the binding grooves of MHC-I molecules. RMSDs of all the complexes were calculated and plotted on the graph shown in Fig. 6. The RMSDs of all of the complexes ranged from 0.08 to 0.2 nm. These results show that small fluctuations were observed during the simulation time, but these fluctuations are likely because some of the peptides are modelled as loop structures.

## Discussion

Vaccination is one of the best options for providing immunity against different pathogenic organisms, thus delivering protection against different diseases. Different types of vaccines, such as peptide, conjugated, subunit, and DNA vaccines, can be used to provoke the immune response. However, in this postgenomic and proteomic era, researchers prefer peptide or subunit vaccines over the whole pathogenic agent due to the availability and accessibility of huge data sets for different pathogens. These data can be systematically analyzed through the use of computational tools. Presentation of MHC-Antigen on the surface of T-cells provide a way to kill the infected cell. This presentation of MHC-antigen complex direct apoptosis or self-eating process of the infected cell. The antigenic element of the pathogen provoke the immune response by activating a signalling process. The peptide fragment bound to MHC molecule is primarily presented T-cell which significantly rely on different factors including proteasome cleavage and transport with the aid of ER. TAP which transporting channels or proteins help in the transport to the surface of the cell. Therefore, considering the c-terminal cleavage activity and TAP efficiency greatly help in the selection of effective vaccine candidates^31,57–59^. Immunoinformatic approaches have contributed greatly to the development of vaccines. Therefore, we employed these tools to design peptide vaccines against EBV to provide a means to protect humanity from the multiple diseases caused by EBV, such as infectious mononucleosis, Burkitt’s lymphoma^60^, Hodgkin’s lymphoma^61^, stomach cancer, laryngeal carcinoma^62^, multiple sclerosis^63,64^ and lymphomatoid granulomatosis^65^. Additional diseases that have been linked to EBV include Giannotti–Crosstie syndrome, erythema multiform, acute genital ulcers, and oral hairy leukoplakia^66^; furthermore, hypersensitivity to mosquito bites has been associated with EBV infection^67^. EBV has been implicated in disorders related to alpha-synuclein aggregation (e.g., Parkinson’s disease, dementia with Lewy bodies, and multiple system atrophy)^68^. The proteome of EBV has many important functional proteins involved not only in its pathogenesis but also in the maintenance of the pathogenic condition. EBV has been reported to use its glycoproteins, such as glycoprotein B, glycoprotein L, glycoprotein H, glycoprotein N, glycoprotein M, glycoprotein 42, and glycoprotein 350, to attach to and infect its host cell. Therefore, predicting and validating both B-cell and T-cell epitopes from these proteins are important steps to provide immunity against these various diseases. Based on MHC binding affinity, TAP score, C-terminal cleavage score, molecular docking and MD simulation, we propose Glycoprotein B (QMDTIYQCY_134–142_), Glycoprotein L (MTAASYARY_256–264_), Glycoprotein N (TTDSEEEIF_396–404_), Glycoprotein M (LTEAQDQFY_43–52_), Glycoprotein 42 (CAELYPCTY_132–140_), and Glycoprotein 350 (PTNTTDITY_316–324_) as the final T-cell epitopes that could provoke the immune response in the host cell. The systems biology approach with pharmacokinetic/dynamics modelling validated that these epitopes significantly activates the immune response pathway and, thus, provide a strong basis for the testing of these epitopes under *in vivo* conditions. Structural stability analysis of the peptide-MHC complexes also revealed the stability of these immunogenic complexes. Antigenic and allergenic profiles also confirmed that these epitopes are strong candidates. Furthermore, B-cell epitopes were reported as the primary choice for the development of a B-cell immune response.

This study provides a means for the development of peptide-based vaccines against EBV infection that could prevent many important diseases. To date, no such computational meta-analysis integrated with dynamics has been reported for the purpose of developing a peptide vaccine for EBV. This multiple step process has noticeably increased the scope and precision of this study. Our results will facilitate efficient subsequent experimental efforts, as the specified regions from Glycoprotein B (QMDTIYQCY_134–142_), Glycoprotein L (MTAASYARY_256–264_), Glycoprotein N (TTDSEEEIF_396–404_), Glycoprotein M (LTEAQDQFY_43–52_), Glycoprotein 42 (CAELYPCTY_132–140_), and Glycoprotein 350 (PTNTTDITY_316–324_) could be used for the development of candidate CTL epitopes. This study will aid in the progress of peptide vaccines against EBV.

## Conclusion

It is well known that EBV causes many human diseases, including cancer. This study integrated multiple approaches to elucidate possible effective peptide vaccines that could provide protection against multiple infections. This study provides insight into the disease-causing factors of EBV virus and, thus, finalized potential B-cell and T-cell epitopes that will aid in the development of effective vaccines. Despite the prediction and validation of such peptides computationally, testing of our predicted epitopes should be carried out in animal models.

## Supplementary information

Supplementary Material
