# Supplementary material for: Immunoinformatic and systems biology approaches to predict and validate peptide vaccines against Epstein–Barr virus (EBV)
Source: Sci Rep. 2019 Jan 24;9:720. doi: 10.1038/s41598-018-37070-z (PMC6346095; doi:10.1038/s41598-018-37070-z)
Supplement: Supplementary file 1 — Supplementary Material [file 41598_2018_37070_MOESM1_ESM.pdf]

Immunoinformatic and systems biology approaches to predict and validate peptide vaccine against Epstein–Barr virus (EBV)

Arif Ali*[1], Abbas Khan[1], Aman Chandra Kaushik[1], Yanjie Wang[1], Syed Shujait Ali[2]. Muhammad Junaid[1], Shoaib Saleem[2], William CS Cho[3], Xueying Mao[4], Dong-Qing Wei[*1]

[1]State Key Laboratory of Microbial Metabolism, and College of Life Sciences and Biotechnology, Shanghai Jiao Tong University, Shanghai, China

[2] Center for Biotechnology and Microbiology, University of Swat, Khyber Pakhtunkhwa, Pakistan

[3]Department of Clinical Oncology, Queen Elizabeth Hospital, Kowloon, Hong Kong

[4]Qianweichang College, Shanghai University, Shanghai China

Dong-Qing Wei[*1], (Corresponding Author) dqwei@sjtu.edu.cn

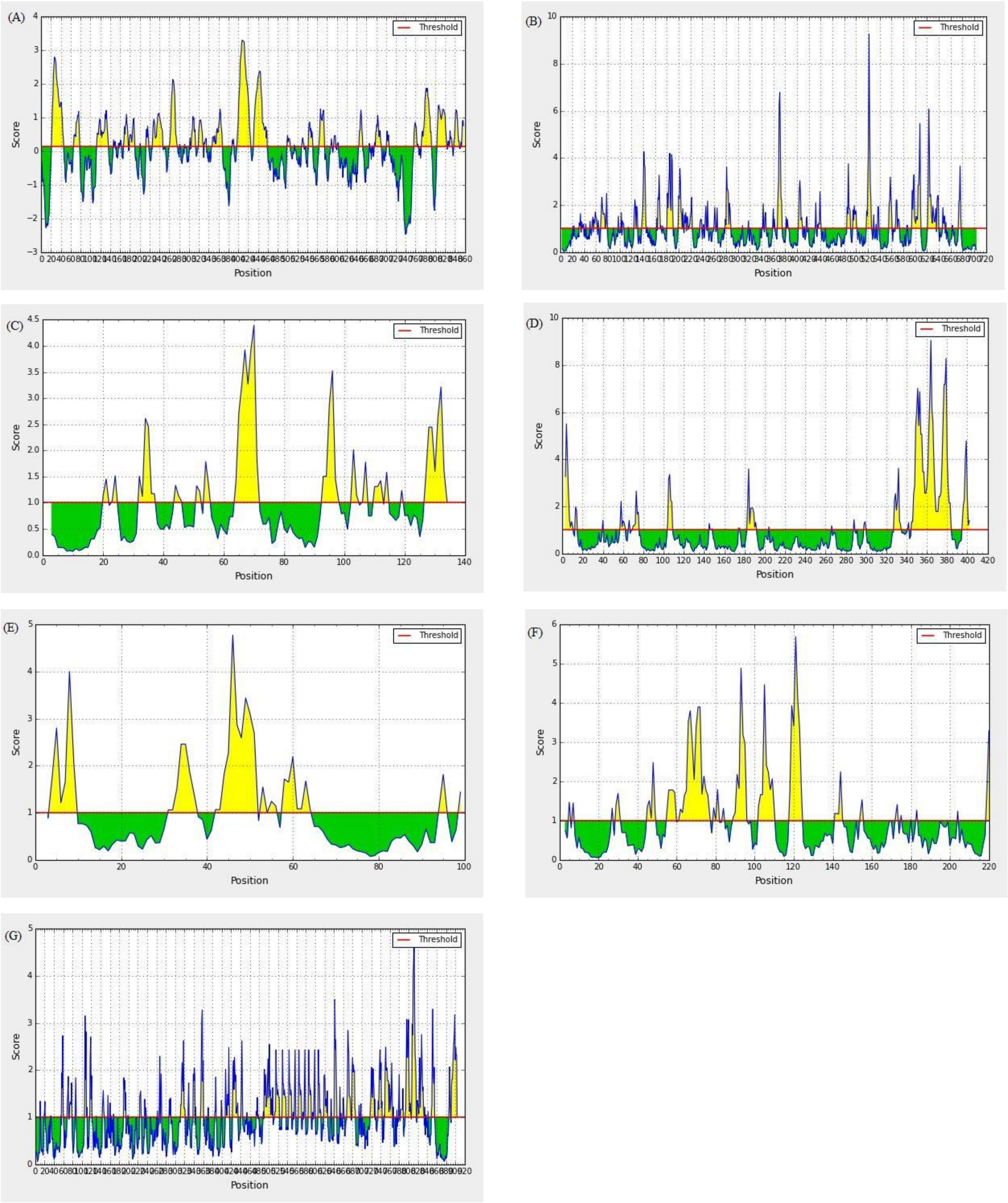

Figure S1: Graphical representation of predicted **Surface accessibility** Epstein-Barr Virus.

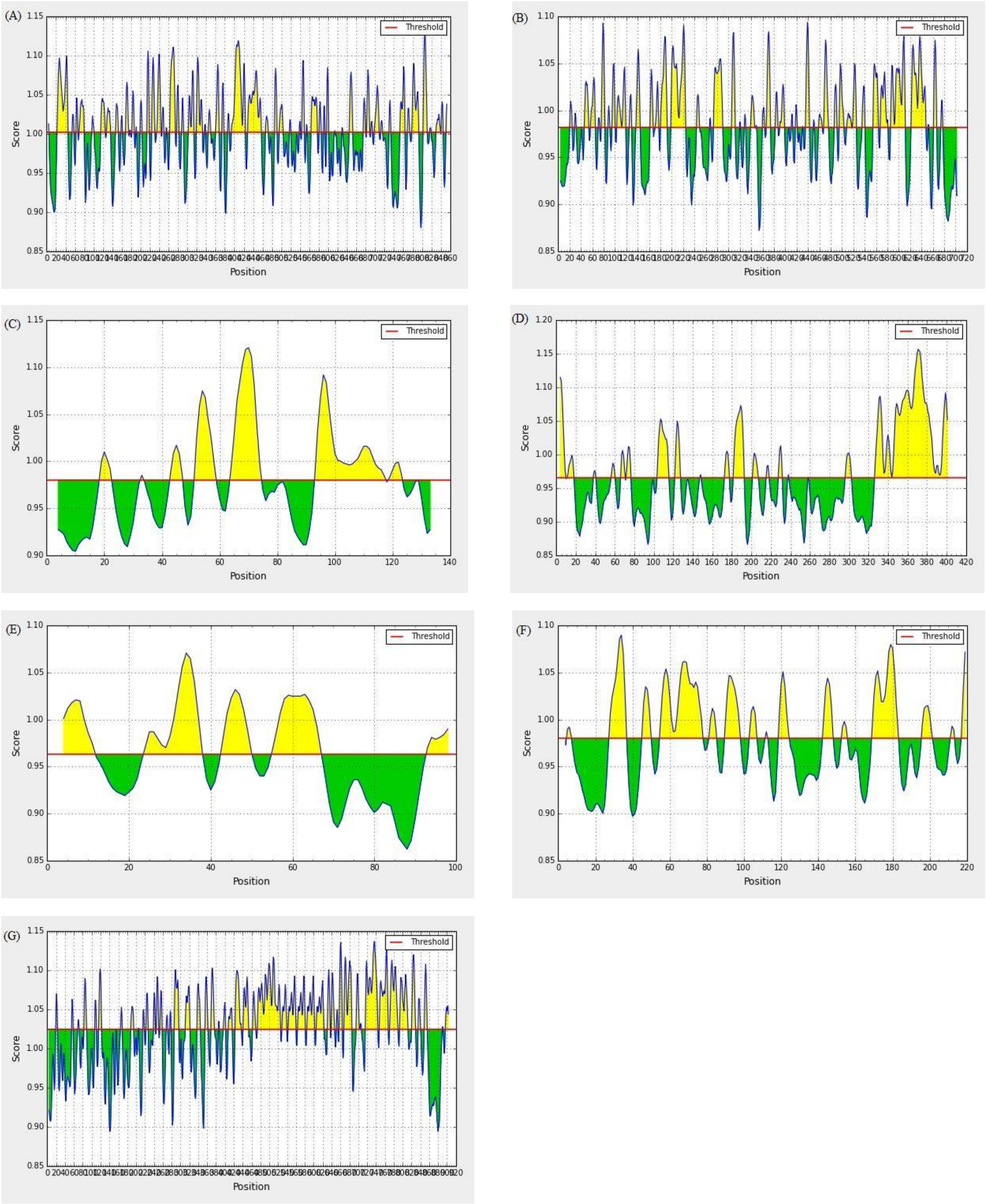

Figure S2: Graphical representation of predicted **Surface flexibility** of Epstein-Barr virus.

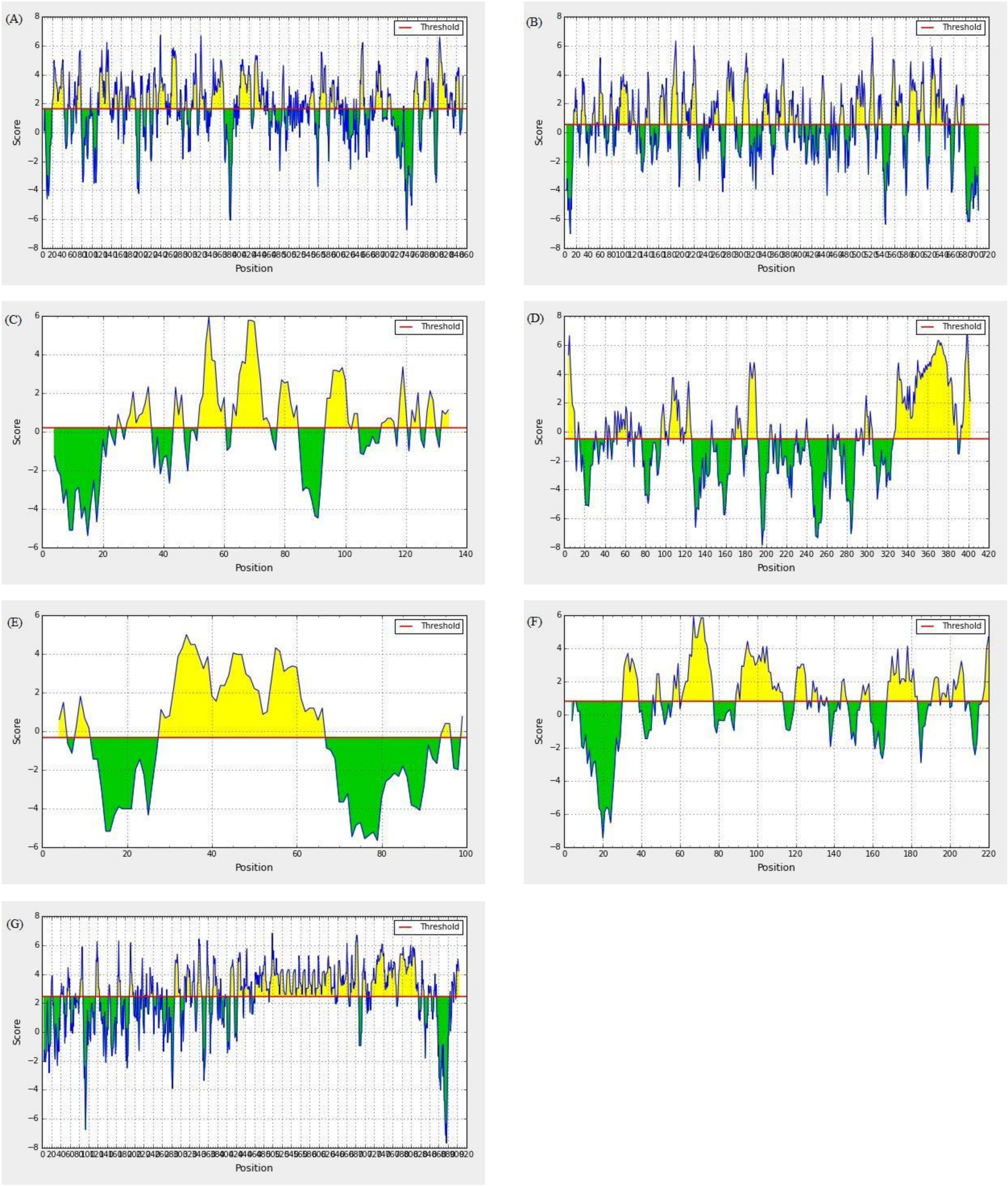

Figure S3: Graphical representation of predicted **Parker Hydrophilicity Prediction for** Epstein -Barr virus.

Table S1: The table is showing the predicted minimum and maximum Surface Accessibility score of selected proteins.

Species	Position	Residue	Start	End	Peptide	Score
**GB**	450	**R**	428	433	RRRRRD	9.415
	747	**I**	745	750	VVILVI	0.032
**GH**	522	**E**	520	525	EREDRD	9.265
	7	**F**	5	10	CVFCLV	0.033
**GL**	70	**G**	68	73	KNGSNQ	4.395
	10	**I**	8	13	LAICLV	0.071
**GM**	364	**R**	362	367	RNRRRS	9.054
	311	**P**	309	314	IIPILC	0.067
**GN**	46	**A**	44	49	TEAQDQ	4.777
	78	**L**	76	81	LVLVII	0.07
**GP42**	121	**K**	119	124	TKKKHT	5.691
	20	**V**	18	23	VIVLLL	0.054
**GP350**	812	**P**	810	815	PRPRYN	4.859
	5	**L**	3	8	AALLVC	0.058

Table S2: The table is showing the predicted minimum and maximum Schulz Flexibility Prediction score of selected proteins.

Species	Position	Residue	Start	End	Peptide	Score
**GB**	806	**Q**	803	809	EQNQEQK	1.13
	798	**M**	795	801	QAIMLAL	0.88
**GH**	439	**Q**	346	442	VITQGPN	1.094
	354	**M**	351	357	LAAMLMA	0.872
**GL**	70	**G**	67	73	PKNGSNQ	1.121
	10	**I**	7	13	FLAICLV	0.904
**GM**	371	**S**	368	374	STSSSSS	1.157
	196	**W**	193	199	FLWWVVF	0.866
**GN**	34	**S**	31	37	GASSPTN	1.071
	88	**M**	85	91	IYLMYVC	0.862
**GP42**	34	**G**	31	37	VRGGGRV	1.09
	40	**A**	37	43	VAAAAIT	0.897
**GP350**	736	**S**	733	739	PGNSSTS	1.137
	880	**M**	877	883	LLVMADC	0.894

Table S3: The table is showing the predicted minimum and maximum Surface Hydrophilicity score of selected proteins.

Species	Position	Residue	Start	End	Peptide	Score
**GB**	240	**N**	237	243	DGKNKET	6.729
	739	**L**	736	742	MLILVLV	-6.743
**GH**	523	**D**	520	526	EREDRDA	6.586
	10	**V**	7	13	FCLVLLW	-7.014
**GL**	55	**T**	52	58	SNQTCDG	5.971
	15	**I**	12	18	LVTIFVL	-5.40
**GM**	399	**S**	396	402	TIDSEEE	7.186
	196	**W**	193	199	FLWWVVF	-7.857
**GN**	34	**S**	31	37	GASSPTN	5.014
	79	**V**	76	82	LVLVIIA	-5.671
**GP42**	67	**A**	64	70	NKTEAQE	5.943
	20	**V**	17	23	LVIVLLL	-7.457
**GP350**	499	**T**	496	502	DNGTESK	6.843
	877	**L**	874	880	LLLLLVM	-7.70
